# AR Signaling in Breast Cancer

**DOI:** 10.3390/cancers9030021

**Published:** 2017-02-24

**Authors:** Bilal Rahim, Ruth O’Regan

**Affiliations:** Department of Medicine, Division of Hematology & Oncology, University of Wisconsin School of Medicine and Public Health, Madison, WI 53792, USA; brahim@uwhealth.org

**Keywords:** AR signaling, AR/PARP interplay, AR/BET interplay, breast cancer

## Abstract

Androgen receptor (AR, a member of the steroid hormone receptor family) status has become increasingly important as both a prognostic marker and potential therapeutic target in breast cancer. AR is expressed in up to 90% of estrogen receptor (ER) positive breast cancer, and to a lesser degree, human epidermal growth factor 2 (HER2) amplified tumors. In the former, AR signaling has been correlated with a better prognosis given its inhibitory activity in estrogen dependent disease, though conversely has also been shown to increase resistance to anti-estrogen therapies such as tamoxifen. AR blockade can mitigate this resistance, and thus serves as a potential target in ER-positive breast cancer. In HER2 amplified breast cancer, studies are somewhat conflicting, though most show either no effect or are associated with poorer survival. Much of the available data on AR signaling is in triple-negative breast cancer (TNBC), which is an aggressive disease with inferior outcomes comparative to other breast cancer subtypes. At present, there are no approved targeted therapies in TNBC, making study of the AR signaling pathway compelling. Gene expression profiling studies have also identified a luminal androgen receptor (LAR) subtype that is dependent on AR signaling in TNBC. Regardless, there seems to be an association between AR expression and improved outcomes in TNBC. Despite lower pathologic complete response (pCR) rates with neoadjuvant therapy, patients with AR-expressing TNBC have been shown to have a better prognosis than those that are AR-negative. Clinical studies targeting AR have shown somewhat promising results. In this paper we review the literature on the biology of AR in breast cancer and its prognostic and predictive roles. We also present our thoughts on therapeutic strategies.

## 1. Introduction

Androgen receptor (AR) signaling has become increasingly important in understanding the biology of breast cancer, and serves as a potential therapeutic target in the era of precision medicine. Previously, breast cancer has been categorized based on hormone receptor (HR) status, and the presence or absence of human epidermal growth factor 2 (HER2) amplification. More recently, it has become apparent that the AR pathway is associated with breast tumor carcinogenesis, with differing mechanisms dependent on co-expression of HR or HER2 amplification [1,2] Although our understanding is still early, this signaling pathway has important prognostic and therapeutic implications. This review will aim to further clarify the complexities of the AR pathway in relation to breast cancer tumorigenesis, prognostic associations in relation to HR expression and HER2 amplification and potential therapeutic options.

## 2. AR Pathway in Breast Cancer

**Figure 1:**
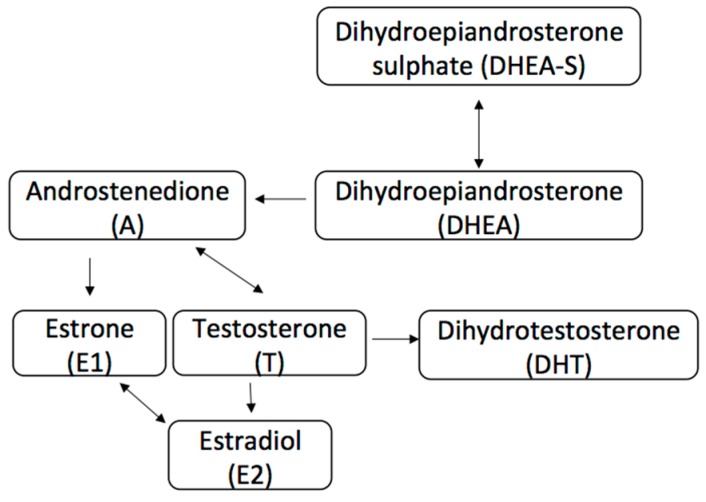
Abbreviated androgen and estrogen pathway. Arrows represent direction of enzymatic conversion.

The AR is a steroid-hormone activated transcription factor belonging to the nuclear receptor superfamily, a group that also includes the estrogen receptor (ER) and progesterone receptor (PR). Upon binding of its androgen ligand, the protein translocates to the nucleus where it stimulates transcription of androgen-responsive genes [2,3]. More recently, non-genomic actions of the AR signaling pathway have been described and are still being investigated in both normal female tissue and in tumor carcinogenesis [4]. AR binds androgens that are produced in a normal physiological manner from the female adrenal glands and ovaries, and in descending order of concentration include dehydroepiandrosterone sulphate (DHEAS), dehydroepiandsoterone (DHEA), androstenedione (A), testosterone (T), and dihydrotestosterone (DHT) (Figure 1) [5,6]. Only testosterone and DHT bind directly to AR, and are primarily formed by peripheral conversion of DHEAS, DHEA, and A in adipose tissue, liver and skin [5,7]. It is important to note that although testosterone can itself bind to AR, or be converted to the more potent DHT via 5α reductase, it can also be converted to estradiol (E2) via the aromatase enzyme that is found in numerous tissues including the breast [8,9,10]. This conversion to estradiol is important, as estradiol serves as the primary ER ligand for both ERα and ERβ receptors in breast cancer. ERα has been shown to have proliferative effect on tumors, while ERβ has been associated with anti-proliferative effect, though these mechanisms are complex and our understanding remains limited [11,12,13,14].

AR is found in up to 70%–90% of all breast cancers, making it more abundant than ER or PR activity [15,16,17,18,19]. However, identifying exact percentages of AR expression among the various breast cancers—HR-positive, HER2-positive, or triple negative breast cancer (TNBC)—is somewhat challenging due to considerable variability in methodology, including differing locations of expression (cytoplasmic versus nuclear), cut off points for immunohistochemical (IHC) receptor expression (≥1%, ≥5% or ≥10%), and the antibody used in staining. One very large systematic review aimed to address AR expression in ER-positive versus ER-negative breast cancers by analyzing 19 studies, including 7693 patients, and found AR co-expression with ER-positive disease to be 74.8% [20]. AR was also found in ER-negative tumors at a lower rate of 31.8% from the same study, although other studies show significant variability in this percentage depending on HER2 or TNBC status [20]. For example, HR-negative and HER2-positive breast cancers seem to express AR in the range of 50%–60%; TNBC is generally between 20% and 40% [15,16,21,22,23,24,25,26,27,28]. AR is also variably expressed in certain histologically distinct subsets of mammary epithelial cells, including invasive apocrine carcinomas, with molecular apocrine cells uniformly expressing AR but not ER or PR [29,30]. Luminal epithelial cells have also been found to express AR in up to 30%, often with co-expression of ER and PR [31].

Gene expression profiling has also led to distinct molecular subtyping that is sometimes used to classify breast cancer, and these tumors seem to show variable rates of AR expression [32,33]. For example, the luminal A and luminal B subtypes, as defined by ER positivity seem to express AR anywhere from 50% to 90% depending on the study. The HER2-positive molecular subtype expresses AR between approximately 20%–60%, and TNBC molecular subtype between 20% and 50% [34,35].

In regards to AR activity in breast cancer carcinogenesis, multiple in vitro studies using several laboratory breast cancer cell lines (i.e., MCF-7, T47-D, and BT20) have shown an anti-proliferative effect of AR antagonism [1,36,37,38,39]. Interestingly, in the presence of ERα, the AR pathway can be either antagonistic or agonistic to tumorigenesis, and at least partially is influenced by the level of receptor expression and availability of their respective ligands [40,41,42,43]. Alternatively, ERα-negative and AR-positive breast cancers fall into a category termed the “molecular apocrine” subtype, with typically distinct histological features of eosinophilic and granular cytoplasm [44]. Within this subtype, a preclinical study by Doane and colleagues utilizing the cell line MDA-MB-453 found the absence of ER but continued dependence on hormonally regulated transcription, which was previously thought to be solely the product of ER activation. Further gene expression profiling revealed the presence of AR, and incubation of the cell line with synthetic androgen led to proliferation that could be blocked by the anti-androgen flutamide [45]. The proliferative activity of AR seems to be consistent in the presence of HER2, with at least partial “cross-talk” between the receptor pathways, and in TNBC as well [46,47,48]. This heterogeneity of AR signaling in relation to co-expression of ERα, HER2 and in TNBC will be discussed in further detail in their respective sections of this paper.

## 3. AR Pathway in ER+ Breast Cancer

Like AR, ER is a steroid hormone receptor. There are naturally two receptors expressed in normal breast tissue, ERα and ERβ, which are involved in the development of reproductive organs, bone density, cell cycle regulation, DNA replication and variety of other processes that occur through both genomic and non-genomic mechanisms. Its ligand is estradiol, and in normal breast tissue ERβ is the dominant receptor. In breast cancer, ERα expression increases and is implicated in tumorigenesis [49]. The function of AR depends largely on the level of co-expression of ERα in HR-positive breast cancer (i.e., luminal breast cancer). Interestingly, many pre-clinical studies show differing proliferative versus anti-proliferative effects in ERα and AR-positive breast cancer that correlates with variation in the ratio of these steroid receptors and the availability of their respective ligands (i.e., estradiol and DHT). As noted earlier in this paper, androgens can be peripherally converted to estradiol (Figure 1), making the interplay between androgens and estrogens in patients expressing both AR and ER quite complex. Early in vitro studies have tried to elucidate the complex relationship between AR and ER expression and the variable responses to hormones and their antagonists in breast cancer cells.

Some studies show AR agonists to actually have anti-tumor effect in the setting of ERα. This has been demonstrated through in vitro modeling in which higher levels of AR confer anti-proliferative effects in the MCF-7 cell line [41]. Some older in vitro studies show increased apoptotic activity with the use of androgens, as well as down regulation of the *bcl-2* proto-oncogene, which could be reversed with the addition of the anti-androgen hydroxyflutamide [50,51]. There are even some older clinical trials that have demonstrated that treatments with exogenous androgens can successfully treat certain breast cancers, with regression rates of approximately 20% [42]. These early clinical studies, though, did not categorize the receptor status of treated patients.

Overexpression of AR in the MCF-7 breast cancer cell line, as postulated by Britton and colleagues, is thought to be due to cross talk between ERα and the EGFR/MAPK pathway, which leads to a self-propogating autocrine growth-regulatory loop through ERα mediated development of AR [52]. Yeast and mammalian two-hybrid systems found ER and AR co-expression led to ER-AR heterodimerization, rather than ER-ER or AR-AR homodimerization, and thus a decrease in AR transactivation by 35% [43]. This fell in line with other older studies, which showed a dose-dependent decrease in AR transcriptional activity in the presence of ER co-expression and estradiol [53]. Another potential way AR down-regulates ERα activity is by competing for and binding to estrogen response elements (EREs) on DNA [54]. Chromatin immunoprecipitation sequencing (ChIP-seq) and gene microarray analysis of the ZR-75-1 luminal breast cancer cell line identified that increased presence of one respective steroid hormone ligand (DHT versus estradiol) over the other leads to antagonism of the other pathway, specifically at the level of transcription by binding to DNA response elements [40]. For example, if AR binds to EREs it leads to an anti-proliferative effect rather than the proliferative effect of ERα binding to ERE and vice versa for ERα binding to androgen response elements (AREs). In certain studies, ER and AR interplay actually leads to increased resistance to traditional endocrine targeted therapies [55,56].

ER expression serves as a primary target for therapy and one of the first treatments targeting this pathway was the anti-estrogen tamoxifen, which was FDA approved in 1998. It is a selective estrogen receptor modulator (SERM) that has differential ER agonist and antagonist activity depending on the target tissue, and acts as a competitive inhibitor of estradiol [57]. Tamoxifen-resistance can occur in HR-positive breast cancers and AR signaling has been implicated in this process, leading to some clinical insight into the relationship between ER and AR signaling pathways. Toth-Fejel and colleagues noted the androgen DHEA-S induced growth in the AR and ER-positive cell line T-47D by 43.4%, but inhibited the AR-positive and ER-negative cell line HCC1937 by 22% [58]. They also found that pre-treatment of the cell lines with tamoxifen in T-47D cells could increase the inhibitory activity of DHEA-S, presumably though increased activity at the level of the AR receptor.

A somewhat conflicting pre-clinical model to that of Toth-Fejel and colleagues noted in the MCF-7 cell line that overexpression of AR made ERα-positive breast cancer cells resistant to the inhibitory effects of tamoxifen in xenograft and nude mice studies, and that treatment with anti-androgen therapy could overcome this resistance [59]. The postulated mechanism was an AR-associated increase in tamoxifen agonist activity on ER, rather than an antagonistic effect [58,59]. A more recent preclinical study found that the agonist activity of tamoxifen on ER signaling in the presence of high levels of AR leads to activation of epidermal growth factor receptor (EGFR), which could be blocked by use of the non-steroidal anti-androgen enzalutamide and/or the anti-EGFR therapy gefitinib [60]. Additionally, tamoxifen-resistant cancers in which AR is present tend to have both higher levels of AR expression and in one study, higher AR to ER nuclear expression [56].

## 4. Prognostic Implications of AR in ER+ Breast Cancer

Several larger studies and meta-analyses reviewing the prognostic implications of AR-positive breast cancer report their findings without discussion of AR in relation to co-receptor status, or male breast cancer. Given the size of these studies, and the unique look into male breast cancer, they remain important and will be discussed briefly here prior to reviewing the significance of AR in relation to ER in this section, and HER2 co-expression versus TNBC in later sections. The largest meta-analysis to date presented by Vera-Badillo and colleagues encompassed 19 studies and 7693 patients with stage I-III disease [20]. Specifically, this study looked at the odds ratios for overall survival (OS) and disease free survival (DFS) at 3 and 5 years for patients with AR expression, in which 4658 patients (60.5%) had breast cancers that were notably AR-positive. Independent of ER expression, patients with AR-positive breast cancers were found to have statistically significant improvements in OS and DFS at both 3 year and 5 year time points, including a 13.5% absolute improvement in 5 year OS and 20.7% in DFS [20]. Another meta-analysis reviewing DFS and OS by Qu et al. evaluated 12 studies and 5270 patients that met their criteria. The combined hazard ratio for DFS of all included studies was 0.52, which was statistically significant, indicating a lower risk of recurrence for patients with AR-positive breast cancers. However, although showing a trend toward improvement, the difference in OS was not statistically significant [61]. Aleskandarany et al. performed a retrospective cohort study of stage I-III patients (*n* = 1141) with tumors ≤5 cm from 1987 to 1997 [62]. High AR expression was associated with longer breast cancer specific survival (BCSS) and was an independent predictor of better outcome regardless of tumor size, grade and nodal stage. Moreover, low AR expression was associated with increased risk of distant metastasis [62]. The Nurses’ Health Study (NHS) showed similar results in a prospective analysis of stage I-III patients conducted from 1976 to 2008 of postmenopausal women. AR-positive tumors were associated with small tumor size (≤2 cm), lower histologic grade, and stage. Breast cancer survival rates at 5 and 10 years were 88% and 82% for AR-negative patients, and 95% and 88% for AR-positive patients [63].

Regarding male breast cancer, these cases comprise only approximately 1% of all breast cancer. In a Chinese study analyzing 116 patients from 1995 to 2008, men were found to have poorer outcomes if their breast cancers were AR-positive [64]. Unlike comparable studies in female breast cancer, AR expression was not correlated with pathologic T stage, histologic grade, or HR expression. Likewise, in contrast to the studies outlined above, OS and DFS rates were significantly shorter with 5 year OS at 54% versus 72%, and 5 year DFS at 39% versus 61%, for AR-positive versus AR-negative cancers respectively, echoing the results of an earlier Polish study [65,66]. However, AR signaling in male breast cancer remains poorly understood with conflicting results, largely due to the relatively small series available. Where one study indicates a lack of correlation between AR expression and male breast cancer, another indicates decreased AR expression is correlated with earlier development of cancer [67,68]. Further studies are necessary to help clarify this patient population further.

In relation to ER-positive breast cancer, several studies have established that AR positivity has prognostic value. AR and ER co-expressing breast cancers generally have better outcomes in terms of time to relapse (TTR), as well as disease specific survival (DSS) as noted from a study by Castellano and colleagues [69]. The study analyzed 953 ER-positive patients from 1998 to 2003 treated with chemotherapy, hormone therapy or both, of which 859 were evaluable for AR expression and 609 were positive (70.9%). The median TTR was 11.72 years versus 13.22 years and the DSS was 12.33 and 13.91 respectively. Regarding clinical and pathologic features, the study established a correlation with AR positivity and smaller tumor size (<2 cm), absence of lymph node metastases and PR expression [69].

A Swedish population-based prospective cohort study assessing patients from 2002 to 2012 also showed a statistically significant improvement in DFS (at 6 years, approximately 90% versus 78%) in breast cancers co-expressing AR and ER [70]. Tsang et al. reviewed data from 3 Chinese institutions from the years 2002 to 2009 and showed AR and ER co-expression to be associated with lower pathologic T stage, lower tumor grade, PR positivity and better outcomes, and postulated that the favorable result could be due to the inhibitory effect of the AR signaling [34,58]. The Nurses’ Health Study noted the best survival rates in AR and ER co-expressing breast cancers were in postmenopausal women with stage I-III breast cancer, with an overall 30% reduction in breast cancer mortality [63]. Jiang and colleagues also noted a significantly better DFS in the ER-positive molecular luminal (A and B) subtypes [71].

Reduced AR expression in ER-positive disease can predict for an increased risk of relapse, breast-cancer associated death and worse DFS as well [71]. A study of 215 invasive ductal carcinoma samples noted that breast cancers with higher expression (median of 75% nuclear positivity by the AR-U407 IHC assay), was associated with a 3 fold increased risk of relapse and 4.6 fold increased risk in breast cancer related death, as well as a statistically significant decrease in OS [54].

## 5. AR Pathway in HER2 Amplified Breast Cancer

The HER2 receptor in breast cancer was first noted in the late 1980s. Historically, it has been associated with poorer outcomes and is amplified in approximately 15%–25% of invasive breast cancers [72,73]. HER2 amplified breast cancers have lower rates of ER co-expression, ranging from 28% to 49%, with typically better outcomes when ER is present [73,74]. Previous molecular studies have distinguished a group of patients with ER-negative but HER2-positive disease that did not easily fall into a pre-defined category. An important study by Farmer et al. in 2005 aimed to better define ER-negative, HER2-positive disease by tissue microarray and found an increase in AR signaling [44]. These cells in further review were notable for apocrine differentiation when exposed to high amounts of androgens in the in vitro setting, and became known as molecular apocrine with separate distinct characteristics than traditional apocrine tumors [44,75]. One early pre-clinical study postulated that the molecular apocrine subtype was associated with cell proliferation in the presence of androgen due to complex interactions between AR and the HER2 signal transduction pathway in the absence of interference by the ER pathway [45]. A related investigation in prostate cancer found that HER2 kinase signaling is required for full activity of AR at low androgen concentration. In particular, HER2 signaling led to increased binding of AR to the appropriate DNA targets to promote transcription, and protected AR from ubiquitin associated degradation [76].

This interplay was further elucidated by Naderi and Hughes-Davies, who showed in the cell lines MDA-MB-453 and MDA-MB-361, and in fresh tumor samples, that there is cross-regulation of certain genes between AR and HER2. In particular, there was increased expression of steroid response genes FOXA1, XBP1 and TFF3, as well as, increased cell proliferation when either AR or HER2 were stimulated. When exposed to the anti-androgen flutamide, or HER2 inhibition there were pro-apoptotic effects, which was notably additive when given in combination [46]. Later, the same group in a study by Chia et al., further identified a positive feedback loop between the AR and extracellular signal-regulated kinase (ERK) signaling pathways, in which HER2 is a transcriptional target of AR, and leads to increased ERK activity [77]. The ERK pathway was also found to increase AR expression, which could be down-regulated both with the androgen targeting flutamide, or the ERK pathway targeting MEK inhibitor in an in vivo mouse model [77]. Similar models have been described in prostate cancer, and serve as potential therapeutic targets [78].

To further add to the complexity of AR in HER2 amplified disease, a study by Ni and colleagues looking at the AR cistrome in the MDA-MB-453 breast cancer cell line, had several interesting findings. They noted that forkhead factor binding motif FOXA1, was highly expressed in HER2 and AR-positive breast tumors, which is similar to AR-positive prostate cancers and seems to be involved in recruitment of ER and AR to their transcription regulatory elements [28,79,80]. AR mediated activation of HER2/HER3 signaling led to increased activity of MYC gene activity, which increased transcriptional activity of androgen-response genes in ER-negative and AR-positive molecular apocrine breast cancers [81]. In an earlier study, this same group showed DHT stimulation, likely through AR promotion of FOXA1 and wnt/B-catenin pathway led to up-regulation of HER2 and HER3 phosphorylation and activation of the phosphoinositide 3-kinse (PI3K) pathway in the MDA-MB-453 cell line.

Also identified by another group is that AR activates the Wnt/β-catenin pathway, which leads to upregulation of HER3 and has been previously implicated in breast oncogenesis [82]. Exposure to the androgen DHT led to increased growth signaling activity of AR, HER2/HER3 and as a downstream event, and activation of PI3K/AKT pathway and these events could be blocked with the addition of the anti-androgen bicalutamide in an in vivo mouse model [28]. It should be noted that the cell line used in this study has been found to have a homozygous deletion of TP53, a homozygous PTEN missense mutation, and an oncogenic mutation in PI3K that might confound this data [83,84,85].

## 6. Prognostic Implications of AR in HER2 Amplified Breast Cancer

The prognostic significance of AR in HER2 amplified breast cancer seems to either show no association with survival, or indicate poorer outcomes. However, many of these studies are limited by smaller sample sizes. One analysis looking at prognostic variables in AR expressing breast cancer showed no association with BCSS or distant metastasis free interval, though this only comprised a sample of 59 patients [62]. A large prospective study assessing postmenopausal women notably had 1154 samples with AR-positive disease, but only 81 patients with HER2 amplification and noted no differences in survival [63].

Other studies, including a retrospective analysis by Park et al. analyzed 931 breast cancer tissue samples in stage I–III disease without prior therapy. Forty-nine patients with AR-positive, HER2 amplified breast cancer were categorized as molecular apocrine subtype, and survival analysis revealed a trend toward poorer OS, though this did not reach statistical significance [23]. Along these lines, Schippinger and colleagues in a study looking at 232 specimens of metastatic breast cancer noted that DFS in patients with AR expression and HER2-amplification was 9.07 months compared to 17.51 in all patients with AR expressed disease, though again not statistically significant. Moreover, the median survival after recurrence (SAR) in this population was only 10.89 months, which was similar to the 11.99 months in patients with AR-negative disease [86].

## 7. AR Pathway in TNBC

TNBC, as defined by lack of expression of ER and PR and a lack of HER2 amplification, comprises between 10% and 20% of all breast cancers [48,87,88]. Traditionally, outcomes in TNBC have been poor with a median overall survival in metastatic disease of approximately 13 months, as well as a shorter time from recurrent disease until death compared to other breast cancers [89,90]. Pathologic features include higher mitotic indices and an increase in BRCA1 mutations [91]. Demographically, TNBC has been associated with higher proportion of African American and Hispanic patients based on population studies and tend to occur at a higher frequency in younger patients [92,93,94,95]. Despite these common characteristics, TNBC remains a biologically variable disease and thus a common signaling pathway that could serve as a target for therapy has proven elusive [96]. Traditional cytotoxic chemotherapy remains the main approach to treatment in these patients, but significant research at the molecular level is being conducted to identify at least subsets of TNBC that might benefit from treatments focused on driver pathways such as AR signaling.

Gene expression profiling has increasingly been used to classify invasive cancer subtypes over the last 15 years. In TNBC, the majority of cases fall into a category of basal-like subtype, first described by Perou, et al. in 2000 [32]. Expanded studies on the basal-like subtype have identified that this heterogeneous group comprises approximately 16% of all breast cancers [97]. The basal-like subtype has several common and more aggressive clinical features, including higher histologic grade and mitotic indices, as well as earlier disease recurrence that lead to poorer outcomes [33,98,99,100]. Many of these features have clinical overlap with the broader category of TNBC. Depending on the study, the basal-like subtype is found in anywhere from 56% to 95% of cases and has sometimes been used synonymously with the term TNBC [101,102,103,104]. With improved methods in molecular biology and gene expression profiling, the heterogeneity of TNBC is becoming increasingly understood.

Lehmann et al. initially categorized TNBC into 6 separate subtypes, including basal-like 1 (BL1), basal-like 2 (BL2), immunomodulatory (IM), mesenchymal (M), mesenchymal stem-like (MSL), and luminal androgen receptor (LAR), each with distinct gene signatures predicting for driver signaling pathways that could potentially serve as therapeutic targets [48]. Specifically, the LAR subtype was found to be enriched in mRNA expression of AR signaling, as well as multiple downstream AR targets with in vitro studies showing increased sensitivity to the AR antagonist bicalutamide [48]. Lehmann and colleagues later adjusted their classification in 2016, utilizing more refined techniques, to include only 4 subtypes with the omission of the IM and MSL categories [105]. Regardless, the LAR subtype remains validated within the Lehmann lab and among other research groups, including Yu et al., and more recently Jezequel and colleagues who found the subtype to account for approximately 22% of TNBC [106,107]. Moreover, Lehmann’s group later noted that all commercially available AR expressing TNBC cell lines also had PIK3CA mutations. They performed Sanger sequencing on 26 AR-positive and 26 AR-negative TNBC clinical cases, and found clonal PIK3CA mutations were significantly higher in AR-positive (40%) versus AR-negative (4%) tumors [108]. Further analysis of 5 LAR cell lines revealed activating PIK3CA mutations and sensitivity to PI3K inhibition suggesting interplay between these pathways as well [48,108].

Even non-LAR TNBC cell lines SUM159PT, HCC1806, BT549, and MDA-MB-231 seem to have a role for AR signaling. Gene microarray and ChIP-seq analysis shows AR mediated up-regulation of the EGFR ligand amphiregulin, which promotes proliferation via the EGFR pathway. This proliferative activity appeared to be blocked with the anti-androgen enzalutamide [47].

## 8. Prognostic Implications of AR in TNBC Breast Cancer

AR positivity has been associated with more favorable prognoses in TNBC. There are several studies that show AR is associated with lower Ki-67 proliferative marker, lower mitotic score, lower histologic grade and lower clinical stage [23,27,63,109,110,111,112]. Interestingly, TNBC has been associated with the poor prognostic TP53 mutation in up to 80% of patients, but at least one study has shown that patients with AR-positive TNBC have a lower rate of TP53 mutations as well [109,113]. This improvement in histological and genetic features seems to translate to clinical benefit and AR-positive TNBC have both improved DFS and OS versus AR-negative [110,114,115] One retrospective study analyzing tissue microarrays from 287 patients with operable TNBC breast cancer found a statistically significant decrease in lymph node positivity in AR-positive disease. The same study showed a significant difference between AR-positive and AR-negative disease in which 5 year DFS was 87% versus 74.2% and 5 year OS was 94.2% versus 82.3% [114]. A prospective study by Loibl and colleagues that was linked to the German GeparTrio trial noted AR expression predicted a significantly better 5 year DFS of 85.7% compared to 65.5% and 5 year OS of 95.2% compared to 76.2% [116]. Other studies have also shown that lack of AR expression is associated with an increased risk of recurrence and distant metastases, especially in patients with lymph node positive disease [111,112].

Other analyses have shown either no difference or worse outcomes for AR-positive TNBC. McGhan et al., looking at 119 patients with resectable disease, found patients with AR-positive cancers trended toward more advanced stages (stage II and III) breast cancer, with no differences in DSS or OS [21]. Mrklic in a retrospective study analyzing 83 patients with TNBC found no difference in DFS and OS in patients with AR-positive disease versus AR-negative, though only 27 cancers were AR-positive [27]. Pistelli in a similar study analyzing 81 cancers with only 15 positive for AR showed no difference in DFS and OS, and the same was the case for Park and colleagues, in which 21 of 156 TNBC samples expressed AR and no survival differences were noted [117,118]. Another study with 97 AR-positive TNBC cases failed to find a difference in relapse free survival (RFS) or OS compared to AR-negative disease [119]. The large prospective NHS study previously referenced was also evaluated for the prognostic significance of AR in TNBC and found that in 78 out of 211 AR-positive TNBC there was a statistically significant 83% increase in overall mortality compared to AR-negative in a multivariate model [63]. This data conflicts with most other studies as noted above, which generally show improved to no differences in outcomes.

More recently, pathological complete response (pCR) has become a surrogate marker for outcome in patients treated with neoadjuvant therapies [120]. In terms of chemosensitivity in AR-positive TNBC, a limited number of studies have shown a lower rate of pCR. Loibl and colleagues in the GeparTrio trial showed AR-positive disease to have a pCR of 12.85% (*n* = 358), compared to AR-negative tumors at 25.4% (*n* = 315). In multivariate analysis, AR independently predicted pCR. Interestingly, though patients with AR-negative disease had a higher chance of achieving pCR, those with AR-positive disease had similar DFS and OS whether or not pCR was achieved [116]. Specifically, patients who achieved a pCR and had AR-negative cancers had a 5 year DFS of 77.9% and 5 year OS of 87% compared to patients who did not achieve a pCR and were AR-positive in which DFS was 77.5% and OS 88.6% [116]. The patients in the GeparTrio trial received a regimen of doxorubicin, cyclophosphamide and docetaxel (TAC), and if considered a non-responder, went on to receive either more TAC or vinorelbine and capecitabine prior to surgical intervention [116]. Another retrospective study by Asano and colleagues examined 177 patients with resectable early stage breast cancer treated with neoadjuvant fluorouracil, epirubicin, and cyclophosphamide (FEC100) followed by paclitaxel. Sixty-one patients were found to have TNBC, with 23 (37.7%) of these with AR positivity. Though the numbers were quite small, the pCR rates were lower in AR-positive versus negative disease at 17.4% (*n* = 4) compared to 63.2% (*n* = 24) [121]. Notably, the latter AR-negative pCR response was particularly robust in comparison to the Loibl study, and likely contributed to improved OS and non-recurrence free survival in AR-negative TNBC in their population [121].

Also, of interest and somewhat opposite to the above studies looking at pCR is a recent article by Jiang and colleagues in which whole exome sequencing was performed on 29 biopsy samples obtained prior to treatment of patients who were found to have either a pCR (*n* = 18) or extensive residual disease (*n* = 11) after neoadjuvant chemotherapy with adriamycin, cyclophosphamide and paclitaxel (ACT). Pathway databases were used to predict the impact of somatic mutations on certain pathways associated with cancer. Though no single mutation was found to be predictive of response to chemotherapy in TNBC, they did find tumors with mutations in the AR pathway and FOXA1 transcription factor networks had a significantly higher pCR (94.1% vs. 16.6%) compared to those that did not carry such mutations [122]. The FOXA1 transcription factor is thought to be activated by AR signaling [123]. It should be noted that the study did not designate if the samples with somatic mutations in the AR or FOXA1 pathways expressed AR by IHC, which is the surrogate marker of AR pathway activity in most studies.

## 9. Treatment Options in AR+ Breast Cancer

### 9.1. Bicalutamide

**Figure 2:**
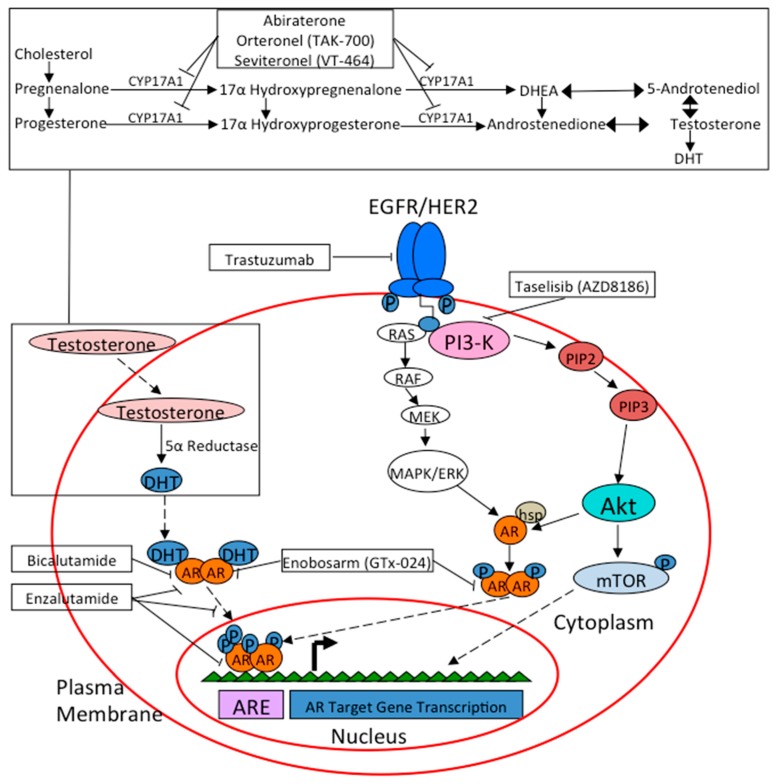
Drug targets in AR signaling pathway.

As previously described, the mechanism of AR-signaling in breast cancer is quite complex and depends on the presence or absence of other signaling mechanisms in concert (Figure 2). Early pre- clinical models have shown both a proliferative effect of androgens on cell activity and an anti-proliferative effect, leading to studying the therapeutic effects of anti-androgen medications. Bicalutamide is a non-steroidal peripherally selective anti-androgen that binds AR as an antagonist [124]. One study showed that in MCF-7 cells transfected with an AR vector, androgens prevented the cells from proliferating, while the addition of the synthetic anti-androgen bicalutamide actually reversed this effect, leading to continued proliferation [41]. Another study by Toth-Fejel and colleagues further differentiated cell lines into ER and AR-positive versus ER-negative and AR-positive disease. They found ER-negative and AR-positive cells were inhibited by 22% with the addition of androgen, but that this could be reversed with pre-treatment with bicalutamide. However, bicalutamide was not studied in the cell line that was ER and AR-positive, thus it was unclear what effect it might have on cell proliferation (i.e., inhibition of cell proliferation?) [58]. De Amicis and colleagues studied the interplay between AR expression and response to the anti-estrogen tamoxifen in the ER and AR-positive MCF-7 cell line. They found in tamoxifen-resistant cells an elevated level of AR and reduced ER mRNA, essentially showing that AR overexpression was associated with tamoxifen resistance, possibly by enhancing its agonistic effects rather than antagonist. This resistance could be overcome with the addition of bicalutamide, which offers interesting therapeutic implications in tamoxifen resistance cancers in which AR is expressed [59]. Further studies assessing bicalutamide in treatment of tamoxifen resistance, or as prophylaxis to resistance, in ER and AR-positive disease are certainly warranted.

There are not many studies that have assessed the role of bicalutamide activity in HER2 amplified disease. Ni and colleagues though, did show an in vivo ability to block stimulation by androgen and induce apoptosis with the use of bicalutamide in ER-negative, AR and HER2-positive breast cancer, giving further evidence of the possible therapeutic effects of anti-androgens in certain AR-positive breast cancers [28].

Bicalutamide has been studied in TNBC. In addition to identifying the molecular LAR subtype, Lehmann and colleagues found this subtype to be quite sensitive to bicalutamide [48]. Zhu and colleagues showed in MSL TNBC cell lines MDA-MB-231 and Hs578T that androgens induce cell proliferation and inhibits apoptosis in vitro and in vivo and that bicalutamide promotes apoptosis, as well as other inhibitory effects [125]. Another study aimed at understanding the interplay between the transcription factor ZEB1, which plays a role in cancer progression by regulating the epithelial to mesenchymal transition (i.e., increased tumor migration and invasion) in breast cancer, and AR signaling in TNBC noted that by inhibiting ZEB1, AR expression was decreased and perhaps more importantly, inhibition of AR signaling with bicalutamide suppressed ZEB1 expression [126]. Mehta and colleagues analyzed the TNBC cell line MDA-MB-453, which in addition to AR positivity, also has PTEN and p53 mutations [127]. They identified 10 genes as AR targets using RT-qPCR and ChIP sequencing techniques and found that androgens promote cell proliferation and decrease apoptosis via these gene targets. They found that the addition of the anti-androgen bicalutamide could reverse this effect. Additionally, they hypothesized that the reason for poorer response to adjuvant or neoadjuvant chemotherapy in AR-positive TNBC was due to an AR-mediated resistance to apoptosis. The effects of paclitaxel, 5-fluorouracil and cyclophosphamide in AR-positive TNBC were studied and cells were found to have significant increases in cell survival and decreased apoptosis in the presence of androgen and that this could be reversed with the addition of bicalutamide [127]. As previously noted, patients with TNBC receiving neoadjuvant chemotherapy have been found to have lower pCR rates when AR-positive and this study provides rationale that perhaps targeting the AR pathway may help improve pCR rates [116].

Evaluation of the correlation between membrane tyrosine kinase receptors and expression of AR in TNBC has shown a positive correlation with EGFR, and platelet derived growth factor beta (PDGFRβ) [128]. The same study found increased PI3K/Akt activity in AR-positive TNBC and found that co administration of bicalutamide with agents targeting EGFR, PDGFRβ, PI3K/ mammalian target of rapamycin (mTOR), and ERK pathways led to synergistic activity and provides some rationale to further evaluate combination therapy in AR-positive TNBC [128]. To further the argument that dual blockade of AR and PI3K/mTOR inhibition can lead to synergistic effects, is a study by Lehmann and colleagues. They noted a much higher rate of concurrent clonal phosphatidylinositol-4,5-bisphosphate 3-kinase, catalytic subunit alpha gene (PIK3CA) mutations (40%) in AR-positive TNBC versus AR-negative (4%), and also that targeting dual targeting of PI3K and AR had an additive inhibitory effect on tumor growth [108].

An alternative combination target may include the use of cyclin-dependent kinases 4 and 6 (CDK4 and CDK6). These kinases are activated by cyclin D, and promote cell cycle entry by phosphorylating proteins that drive the transition from G1 to the S1 phase and when disrupted can lead to unrestricted cell proliferation in breast cancer [129,130]. Certain preclinical models have shown that resistance to anti-androgen therapy is linked to a F876L mutation in AR, leading to a change from antagonist activity to agonist. CDK4/6 inhibitors have been shown to restore activity of anti-androgen treatment by antagonizing AR F876L [131]. There is currently a phase I/II trial of palbociclib in combination with bicalutamide for the treatment of metastatic AR-positive TNBC which is accruing (NCT02605486) (Table 1) [132].

Preclinical studies led to a phase II clinical trial evaluating bicalutamide in metastatic ER-negative and AR-positive cancers as a proof of concept study led by Gucalp and colleagues. Patients with >10% nuclear expression of AR by IHC were included and treated with bicalutamide 150 mg daily, with the primary endpoint being clinical benefit rate (CBR) defined as the total number of patients who showed a complete response (CR), partial response (PR) or stable disease (SD) > 6 months. The study found the CBR to be 19% for the 26 study participants, driven by SD as there were no CRs or PRs, and a median progression free survival (PFS) of 12 weeks. Though HER2 status was not an exclusion criteria, only 1 of the 26 patients had HER2 amplified cancers and 1 of the 5 patients with SD had initial negative HER2 status that was later considered positive after undergoing a curative intent mastectomy [133]. A more recent case reported by Arce-Salinas of a patient with recurrent AR-positive metastatic TNBC, molecular apocrine subtype, had a CR with use of bicalutamide despite heavy pretreatment with palliative chemotherapy, showing that a CR with anti-androgen therapy alone does seem to be possible [134]. Briefly, it should be noted that the older nonsteroidal anti-androgen flutamide, which is less potent than bicalutamide, was studied in two phase II clinical trials in 1988 in patients with metastatic breast cancer. Neither of these studies yielded promising results, though were conducted in a patient population unselected for AR, ER, PR or HER2 status [135,136].

### 9.2. Enzalutamide

Enzalutamide is a newer generation nonsteroidal anti-androgen that binds the androgen receptor with greater affinity than bicalutamide, decreases nuclear translocation, and impairs binding to androgen response elements and co-activators [137]. An interesting study by Cochrane and colleagues examined the effects of enzalutamide in AR-positive breast cancer in both ER-positive and ER-negative tumors. The study found both in vitro and in vivo that enzalutamide inhibits androgen mediated growth in both ER-positive and ER-negative cancers expressing AR. Interestingly, enzalutamide also inhibited estrogen-mediated growth in ER-positive, AR-positive cells, whereas previous preclinical studies have shown bicalutamide to increase cell proliferation in this cell population [56]. A more recent study by D’Amato and colleagues had similar results, and found AR inhibition reduced estradiol mediated proliferation in ER-positive and AR-positive disease [138]. These studies suggest that the AR signaling pathway may be a potential target in ER-positive disease as well, which has not been shown with bicalutamide. Along these lines, a number of studies have shown that when ER is expressed in breast cancer, AR positivity is associated with tamoxifen-resistance. Ciupek and colleagues suggest that in the presence of AR, tamoxifen leads to AR-mediated EGFR activation as a mechanism of resistance. This could be blocked with the use of enzalutamide and the EGFR inhibitor gefitinib and may provide a viable preventive or salvage therapy in ER-positive, AR-positive disease treated with tamoxifen [60].

In TNBC, an in vivo study by Barton and colleagues analyzed 4 TNBC cell lines (SUM159PT, HC1806, BT549, and MDA-MB-231) and noted that the anti-androgen enzalutamide was not only active in the LAR molecular subtype, but also in the M, MSL and BL2 subtypes. They noted that AR activation up-regulates the EGFR pathway, as in ER-positive disease noted above, which could be blocked by enzalutamide and makes it potentially applicable to a broader range of TNBC [47]. Combination therapy with anti-androgens and mTOR inhibition has shown some promising results and Robles and colleagues found additive anti-proliferative effect in the LAR molecular subtype in the MDA-MB-453 cell line and LAR xenograft model [139]. Given that mTOR is downstream from PI3K, this further strengthens the rationale that the PI3K is important in TNBC and a possible target with concurrent enzalutamide as well. There is currently a phase IB/II clinical trial that is in process, which is assessing the CBR at 16 weeks of the PI3K inhibitor taselisib in combination with enzalutamide in advanced TNBC (NCT02457910) [140].

Also of importance is that enzalutamide has been associated with immunogenic modulation, which may increase the susceptibility of tumor cells to immune-mediated cell death [141]. A study by Kwilas et al. showed growth inhibition with enzalutamide and abiraterone in breast cancer cells, with improved immune mediated lysis. They found this increase in immune mediated activity to be associated with increased cell surface expression of tumor necrosis factor-related apoptosis-inducing ligand (TRAIL) and reduction in expression of osteoprotegerin (OPG) [142]. Enzalutamide and the anti-androgen abiraterone acetate, which inhibits the CYP17A1 enzyme involved in androgen biosynthesis, decreased cell proliferation and enhanced immune mediated lysis in AR-positive disease. Even more interesting, both of these medications enhanced immune mediated lysis even in AR-negative disease [142]. An earlier study by Kwilas and colleagues also showed increased immune activity when a pox viral based cancer vaccine was combined with enzalutamide in in vivo mice models, and furthers the idea that this medication increases immunogenic modulation and may have importance in newer immunotherapy trials [143]. There is currently an ongoing phase II clinical trial evaluating dual therapy with enzalutamide and the monoclonal antibody trastuzumab in HER2 amplified, AR-positive metastatic breast cancer with a primary endpoint of CBR at ≥24 weeks (NCT02091960) [144]. Although it would be difficult to tease out the immunogenic modulation of enzalutamide in this study, it may boost the effect of trastuzumab. Enzalutamide is also currently being assessed in several AR-positive TNBC clinical trials, either alone or in combination, which will be discussed below.

Traina and colleagues shared preliminary results of a phase II clinical trial assessing enzalutamide in AR-positive metastatic TNBC [145]. The single-arm, non-randomized phase II trial assessed patients with TNBC who screened for AR positivity as defined by AR expression greater than 0% by IHC. A total of 118 women were enrolled in the trial, with a majority of patients treated in the first or second line setting. The primary end point was CBR at 4 months, which was 35% at that time point, and 29% at 6 months. The median PFS was 14 weeks, and included 2 CRs and 5 PRs and the medication was well tolerated without any new safety concerns [145]. As a side benefit, the study also led to the development of a predictive assay termed PREDICT AR, in which they noted patients who responded to enzalutamide had a distinct gene expression profile, and had a better CBR of 36% at 24 weeks compared to 6% in patients who were PREDICT AR-negative [145,146].

As previously discussed, patients with AR-positive TNBC have a relatively low pCR rate of 12.85% [116]. Aimed at this group is a phase IIB clinical trial in the neoadjuvant setting looking at the use of enzalutamide with weekly paclitaxel with a primary endpoint of pCR that is meant to hopefully improve the response rate (NCT02689427) [147]. There is also a feasibility study accruing that is looking at the use of 1 year of adjuvant enzalutamide for the treatment of patients with early stage, AR-positive TNBC (NCT02750358) [148].

### 9.3. Abiraterone

Abiraterone acetate is a selective, irreversible and potent inhibitor of 17α-hydroxylase and 17,20-lyase (CYP17) enzymatic activity and is commonly used in castration-resistant prostate cancer (CRPC) [149]. It has also been studied in ER-positive metastatic breast cancer with at least part of the rationale being that CYP17 inhibition decrease the synthesis of both androgens and estrogens and may be more effective than an AI alone. A phase II, randomized open-label clinical trial assessing 297 patients with metastatic ER-positive breast cancer looked to clarify the role of abiraterone, though AR positivity was not a stratification factor. Eligibility required sensitivity to an aromatase inhibitor (AI) prior to disease progression and AR positivity was reportedly balanced between treatment arms, including abiraterone plus prednisone, versus abiraterone with exemestane versus exemestane alone with primary end point of PFS. Abiraterone either in combination with prednisone or with exemestane did not improve PFS, compared to exemestane [150,151]. Another phase II clinical trial assessed the safety and efficacy of abiraterone plus prednisone in molecular apocrine AR-positive metastatic breast cancer with a primary endpoint of CBR at 6 months. The CBR was found to be 20%, which included 1 CR and 5 SD, although the overall response rate was only 6.7% with a median PFS 2.8 months [152]. At the time of analysis, five patients remained on treatment with clinical benefit ranging between 6.4 and 24 months. There are currently two other clinical trials assessing abiraterone in breast cancer. A phase I/II UK study evaluated abiraterone in postmenopausal women with advanced metastatic ER or AR-positive breast cancer. This study is no longer recruiting, and results are awaited (NCT0075585) [153]. A phase I, open-label, multicenter trial evaluating abiraterone in combination with the PI3K inhibitor AZD8186 in a variety of solid malignancies, including TNBC, is still recruiting patients (NCT01884285) [154].

### 9.4. Newer Anti-Androgens

A number of other novel nonsteroidal anti-androgen agents are currently under analysis. Orteronel (TAK-700) is a reversible, selective CYP17 inhibitor, similar to abiraterone with a higher specificity for 17,20 lyase inhibition and known activity in CRPC [155,156]. This agent is being studied in a phase II clinical trial in patients with AR-positive metastatic breast cancer, with 2 separate cohorts assessing ER-positive disease and TNBC (NCT01990209) [157]. Seviteronel (VT-464) is a similar newer generation CYP17 inhibitor, with a current phase I/II study accruing patients with advanced breast cancer with separate cohorts for ER-positive disease and TNBC (NCT02580448) [158]. There are more potent and novel anti-androgens in development. A recent study by Kandil and colleagues showed up to 30 to 50 fold improvement in activity with the use of pure novel AR antagonists with 7-substituted umbelliferone derivatives over enzalutamide and bicalutamide respectively [159]. These agents clearly require further testing, but purer compounds may be important in AR-positive TNBC in the future if current clinical trials confirm a significant signal.

### 9.5. SARMs

Somewhat contradictory to other studies presenting therapeutic options, Narayanan and colleagues demonstrated in the MDA-MB-231 cell line that nonsteroidal, tissue selective androgen receptor modulators (SARMs), rather than anti-androgens could inhibit breast cancer growth [160]. They chose the genomically stable MDA-MB-231 TNBC cell line, in which they transfected an AR plasmid, over the often used MDA-MB-453 cell line as the latter is known to express mutated AR, PTEN and p53 that could potentially confound results. Both in vitro and in vivo, they found the addition of SARMs inhibited intratumoral expression of genetic pathways that promote breast cancer development, metastasis-promoting paracrine factors (i.e., IL6, MMP13) and cell proliferation [160]. Based largely on this study, a phase II, multicenter clinical trial investigating the efficacy and safety of the SARM enobosarm (GTx-024) in advanced AR-positive TNBC is currently underway (NCT02368691) [161].

### 9.6. Other Drugs

Poly ADP-ribose polymerase (PARP) inhibitors are a group of agents aimed at the PARP1 protein that acts to repair single strand breaks in DNA. These breaks occur frequently in the cell cycle, and rely on mechanisms such as PARP1 activity to resolve the errors via the base excision repair pathway. Patients with breast cancer susceptibility gene 1 (BRCA1) and 2 (BRCA2), as well as partner and localizer of BRCA2 (PALB2) mutations are susceptible to DNA double strand breaks, as these genes normally function to correct such breaks. In patients with these underlying mutations, the addition of a PARP inhibitor leads to cell death due to dysfunction of both repair pathways [162]. In regards to AR signaling and PARP inhibition, there is minimal data. However, Park and colleagues identified that BRCA1 increased ligand-dependent AR transactivation, as well as synergistically combined with co-activators of the AR pathway, leading to increased efficacy. They postulated that lack of the BRCA1 gene would reduce AR-dependent signaling [163]. Shin et al. found non-mutated BRCA2 synergizes with the co-activator p160 to enhance AR-mediated transcription, similar to BRCA1, and was associated with an anti-proliferative effect [164]. A small study evaluated 41 patients with BRCA1 mutations and 14 with BRCA2 mutations and analyzed AR status by IHC and found only 12% of BRCA1, and 50% of BRCA2 mutated tumors expressed AR [165]. Another study found AR positivity in 13 of 43 (30%) BRCA1 and 14 of 18 (78%) of BRCA2 mutated tumors [166]. At present, there have been no preclinical or clinical studies looking at PARP inhibition specifically in AR-positive disease. Although PARP inhibition has become an important tool in breast cancer treatment, especially in BRCA1, BRCA2 or PALB2 mutated cells, its activity needs to be better defined in relation to the AR pathway in preclinical models before we can identify if there is significant rationale for their use in AR-positive disease.

Bromodomain and extraterminal (BET) signaling has emerged recently as an important pathway in AR signaling. These proteins, which are expressed by the majority of cancer cells, are involved in epigenetic activity and chromatin “reading” and include BRD2, BRD3, BRD4 and BRDT [167]. BRD4 has a significant role in RNA polymerase II transcription by helping to recruit the positive transcription elongation factor P-TEFb [168,169]. Previous studies established the anti-cancer activity of BET inhibitors that target BRD4, which was further evaluated in CRPC by Asangani and colleagues [167]. They found BET inhibition with the small molecule JQ1 to induce G0-G1 cell cycle arrest, apoptosis and transcriptional down-regulation of anti-apoptotic BCL-xl in AR-positive cells. Moreover, they noted a direct AR-BRD4 interaction, which was inhibited by JQ1 leading to a more robust anti-proliferative effect than enzalutamide [167].

BET signaling has been studied in breast cancer as well. The ER-positive MCF-7 breast cancer cell was noted to have increased T-bet activity associated with insulin exposure, which also was associated with tamoxifen-resistance [170]. Feng and colleagues furthered this understanding by noting that ER signaling was positively associated with WHSC1, a histone methyltransferase recruited to the ERα gene by BET proteins. They found this pathway could be blocked with BET inhibition with JQ1 and overcome tamoxifen-resistance in cell culture and xenograft models [171]. Further, Sengupta et al. noted JQ1 suppression of estrogen-induced growth and transcription in MCF7 and T47D cell lines [172]. BET signaling has been studied in HER2 amplified breast cancer, using the cell lines HCC1954 and MD-MBA-361 in which it was shown that BET inhibition could overcome lapatinib resistance associated with kinome reprogramming [173]. Other studies have found that resistance to PI3K inhibitors and mTOR inhibitors is associated with feedback activation of tyrosine kinase receptors in metastatic breast cancer and can be overcome with dual use of PI3K and BET inhibition or mTOR and BET inhibition [174,175]. Borbely and colleagues noted activity of combination therapy with a histone deacetylase (HDAC) inhibitor and BET inhibitor JQ1 by increasing activity of ubiquitin-specific protease 17 (USP17), which down-regulated the Ras/MAPK pathway and thus reduced cell proliferation in 2 separate TNBC (MDA-MB-231 and BT549) and 2 ER-positive (MCF7 and T47D) cell lines [176]. Synergy with the chemotherapeutic agents docetaxel, vinorelbine, cisplatin and carboplatin has been shown with JQ1 in preclinical evaluation of several breast cancer cell lines [177]. An association with hypoxia responsive genes and angiogenesis has been noted, which can be down-regulated with BET inhibition in cell culture and xenograft models [178]. Finally, Sahini and colleagues noted that BET inhibition results in growth suppression of TNBC independent of their intrinsic molecular subtype [179]. BET signaling certainly is an exciting area in breast cancer. However, a limitation to all the above mentioned studies regarding BET and breast cancer is that none of them further clarify the role of AR signaling in the effects that are being described. Given the findings in prostate cancer showing clear activity with AR signaling and the BET pathway, it is important to clarify the role of AR and BET signaling in breast cancer in order to identify its role as a therapeutic target. Currently, there are three early phase clinical trials assessing BET inhibitors in TNBC along with other malignancies [180,181,182].

## 10. Discussion

Our study aimed to describe advances in understanding of the complex AR signaling pathways in relation to co-receptor signaling, as well as prognostic and therapeutic implications. However, there are some inherent limitations to the data presented. In particular, several of the above-mentioned pre-clinical studies utilize commercially available breast cancer cell lines. Though there are advantages to the use of these classic cell line models, over time multiple cycles of cell cultures can select for certain subclones that can create variability in genetic and phenotypic expression across labs [183]. For example, in one study the cell line MDA-MB-453 notably had a homozygous deletion in TP53, a homozygous PTEN missense mutation and a PI3K mutation and it is unclear if these are preserved changes in the cell line or unique to the specific version from that particular lab [127]. Several studies do utilize cells fresh tumor samples to help corroborate their findings, but many do not and thus reproducibility of the findings is a question.

Additionally, most in vitro studies do not distinguish whether the cell line, or cells from fresh biopsy material are early stage or metastatic in origin. Independent review of commercially available cell lines reveals that most are metastatic in origin, and often from malignant pleural fluid, which some might argue indicates particularly aggressive biology that does not reflect the general population [183]. Lobular carcinoma represents approximately 10% of all invasive breast cancer, and none of the above studies looking at AR signaling studied these tumors, raising concerns of the generalizability of findings in these patients. In terms of co-receptor expression, ERα is known to be proliferative in breast cancer but ERβ is less understood, especially in relation to AR. It is possible that ERβ, as another steroid receptor, might have importance given the competitive activity between AR and ER as steroid hormones. There is also controversy over what constitutes IHC positivity of AR expression, with cut off values of ≥1%, ≥5% or ≥10% depending on the study. This lack of consensus guidelines makes it difficult to interpret prognostic value of AR expression in comparison between studies. Lastly, several of the larger studies and meta-analyses do not distinguish differences in prognostic value of AR in relation to co-receptor expression of ER, HER2 or in TNBC. These are somewhat offset by the multiple studies reviewed that do distinguish between these different subtypes of breast cancer.

AR remains an area of study that is rapidly evolving. The current study is a comprehensive review of the available data regarding the pathophysiology of AR-positive breast cancer, and makes important efforts to discuss the nuanced differences between AR-positive breast cancers in relation to co-receptor status. Also, prognostic implications of AR are discussed in the same manner, noting clear differences in ER-positive, HER2 amplified and TNBC. Therapeutic targets along the AR pathway are discussed with emphasis on novel agents and combination therapy with promising results. As our understanding of the complexities of AR signaling in regards to tumorigenesis becomes more refined, we will better be able to use AR expression as a prognostic marker and therapeutic target.

## 11. Conclusions

The identification of the AR signaling pathway in breast cancer has led to an interesting and growing field, especially in regards to basic and translational research. Not only have we identified important prognostic associations with ER-positive, HER2 amplified and TNBC, but also potential therapeutic targets either with monotherapy or in unique combinations. Clearly, there is still significant room to expand the field and grow our understanding of these complex pathways, but early work is encouraging regarding the ability to use targeted therapies in new and exciting ways and we look forward to future of the field.

## Figures and Tables

**Table 1:** Ongoing breast cancer clinical trials.

Trial ID	Agent(s)	Mechanism(s) of Action	Patient Population	Study Design
NCT02605486	Palbociclib & Bicalutamide	CD4/CD6 Inhibitor & Androgen Receptor Inhibitor	AR-positive metastatic breast cancer	Non-randomized, open-label, phase I/II
NCT02457910	Taselisib & Enzalutamide	PI3K Inhibitor & Androgen Receptor Inhibitor	AR-positive metastatic TNBC	Partially-randomized, open-label phase IB/II
NCT02091960	Enzalutamide & Trastuzumab	Androgen Receptor Inhibitor & HER2 Targeted Monoclonal Antibody	AR-positive, HER2 amplified metastatic or locally advanced breast cancer	Non-randomized, open label, phase II
NCT02689427	Enzalutamide & Paclitaxel	Androgen Receptor Inhibitor & Microtubule Stabilizer	AR-positive TNBC, stage I–III breast cancer (neoadjuvant therapy)	Non-randomized, open label, phase IIB
NCT02750358	Enzalutamide	Androgen Receptor Inhibitor	AR-positive TNBC, stage I–III breast cancer (adjuvant therapy)	Non-randomized, open-label, feasibility study
NCT00755885	Abiraterone Acetate	CYP17 Inhibitor	ER or AR-positive postmenopausal metastatic or locally advanced breast cancer	Non-randomized, open-label, phase I/II
NCT01884285	AZD8186 +/− Abiraterone Acetate or AZD2014	PI3K Inhibitor +/− CYP17 Inhibitor or mTOR Inhibitor	Advanced TNBC	Non-randomized, open-label, phase I
NCT01990209	Orteronel	CYP17 Inhibitor	AR-positive metastatic breast cancer	Non-randomized, open-label, phase II
NCT02580448	VT-464	CYP17 Inhibitor	Advanced breast cancer. Phase I: TNBC or ER-positive, HER2 negative	Non-randomized, open-label, phase I/II
			Phase II: AR-positive TNBC or ER-positive, HER2 negative	
NCT02368691	GTx-024	Selective Androgen Receptor Modulator	AR-positive advanced TNBC	Non-randomized, open-label, phase II
